# Prevalence and Indicators of Viral Suppression Among Persons with Diagnosed HIV Infection Retained in Care — Georgia, 2010

**Published:** 2014-01-24

**Authors:** Laura Edison, Denise Hughes, Cherie Drenzek, Jane Kelly

**Affiliations:** 1 EIS officer; 2 Georgia Department of Public Health; 3 Division of HIV/AIDS Prevention, National Center for HIV/AIDS, Viral Hepatitis, STD, and TB Prevention, CDC

Advances in treatment have led to dramatic improvements in the health of persons infected with human immunodeficiency virus (HIV). Moreover, treatment can reduce HIV transmission because suppressed levels of circulating virus makes HIV-infected persons less infectious (1). Until recently, antiretroviral therapy (ART) was recommended only for HIV patients with advanced disease (stages 2 and 3), and was optional for patients with early disease (stage 1). In March 2012, national HIV treatment guidelines were changed to recommend ART at all disease stages (1). To establish a baseline for care and treatment outcomes among persons with HIV, the Georgia Department of Public Health (DPH) examined whether viral suppression among HIV patients in Georgia varied by disease stage at diagnosis before implementation of the new guidelines. Disease stage at diagnosis was assessed as an indicator of viral suppression several months after diagnosis, adjusting for age, sex, and race/ethnicity among patients who were reported to DPH with HIV infections newly diagnosed during 2010 and retained in care. This report describes the results of that analysis, which indicated that disease stage at diagnosis was a significant indicator of viral suppression; viral suppression was significantly less frequent among persons with earlier disease stage at diagnosis. Compared with viral suppression among 80.5% of persons with stage 3 HIV disease, only 72.3% with stage 2 disease (prevalence ratio [PR] = 0.9; 95% confidence interval [CI] = 0.8–1.0) and 64.5% with stage 1 disease (PR = 0.8; CI = 0.7–0.9) met criteria for viral suppression, likely resulting from lack of initiating treatment or inadequate adherence to treatment regimens, as suggested in previous studies (1,2). These data can serve as a baseline to determine the impact of the guideline change in the future, and can be used to emphasize the importance of implementing the guidelines by expanding treatment to persons at all disease stages to reach the goal of viral suppression for all persons with HIV, thus closing the gap in viral suppression among persons diagnosed at disease stages 1 and 2. Health-care providers and community-based organizations should inform patients of the recommendation for ART initiation at all disease stages.

Georgia state law* requires that health-care providers report cases of HIV infection and that laboratories report test results indicative of HIV infection (including positive Western blots, all viral loads, CD4+ counts, and viral nucleotide sequence results) to DPH. Prevalence of viral suppression was determined among Georgia patients aged ≥13 years who had HIV infection newly diagnosed during 2010 and who were retained in care by using the last viral load reported at 4–15 months after diagnosis. Patients were considered retained in care if they had at least two laboratory reports containing a CD4+ or viral load >3 months apart during the 4–15 months after diagnosis. Patients who were retained in care and died ≤15 months after diagnosis were included, and 16 patients with no record of their sex at birth were excluded from the analysis.

Viral suppression was defined as a viral load <200 copies/mL as measured using the last viral load reported at 4–15 months after diagnosis. Disease stage at diagnosis was determined by using the first recorded CD4+ count (or percentage of total lymphocytes if CD4+ count was unavailable) ≤3 months after diagnosis and was defined as stage 1 (≥500 cells/*μ*L or ≥29%), stage 2 (200–499 cells/*μ*L or 14%–28%), or stage 3 (<200 cells/*μ*L or <14%). Transmission categories (i.e., male-to-male sexual contact, injection drug use, male-to-male sexual contact and injection drug use, heterosexual contact, and other/unknown) were assigned by reviewing each patient’s HIV infection risk factors and using a hierarchy of risks previously described to determine the most likely route of HIV transmission (3). Missing transmission category data were estimated by using a multiple imputation method, as previously described (3). Because transmission category was not a significant effect modifier or confounder of the association between viral suppression and any other variable, it was excluded from the final model. PRs for viral suppression and 95% CIs were estimated by using log-binomial regression; sex, race/ethnicity, age at diagnosis, and disease stage at diagnosis were included in the model.

During 2010, a total of 2,921 new HIV infections were diagnosed among persons in Georgia aged ≥13 years, 1,340 (45.9%) patients were retained in care, and 958 (32.8%) met criteria for viral suppression. The analysis presented in this report examines the cross-section of the newly diagnosed population that is retained in care. Among those retained in care, the majority were black (53.7%), and 27.3% were aged 25–34 years; 22.9% were aged 35–44 years, and 21.6% were aged 45–54 years. Male-to-male sexual contact (61.7% of men) and heterosexual contact (67.9% of women) were the most commonly reported transmission categories among men and women, respectively (Table 1).

Among the 1,340 persons retained in care, 958 (71.5%) met criteria for viral suppression ≤15 months after diagnosis. Blacks (63.3%) and persons aged 13–24 years (56.5%) had the lowest prevalence of meeting criteria for viral suppression. A lower percentage of persons with stage 1 disease at diagnosis (64.5%) met criteria for viral suppression than those with stage 2 (72.3%), stage 3 (80.5%), or unknown disease stage (66.3%) at diagnosis. By transmission category, the lowest percentage of viral suppression was among men with infection attributed to male-to-male sexual contact and injection drug use (65.3%) and women with heterosexual contact (67.9%) (Table 1).

Race/ethnicity, age, and disease stage at diagnosis were statistically significant indicators of viral suppression ≤15 months after diagnosis: black persons (PR = 0.9; CI = 0.8–0.9), persons aged 13–24 years (PR = 0.8; CI = 0.7–0.9), and persons diagnosed at disease stage 1 (PR = 0.8; CI = 0.7–0.9) and stage 2 (PR = 0.9; CI = 0.8–1.0) had a lower prevalence of viral suppression, compared with white persons, persons aged ≥55 years, and persons diagnosed at disease stage 3, respectively (Table 2).

## Editorial Note

Efforts are ongoing on national and local levels to promote HIV testing, identify those with acute infection, link and retain persons living with HIV infection in medical care, and achieve higher rates of viral suppression. Monitoring these steps throughout HIV diagnosis and treatment, known as the HIV care continuum, can be used to assess progress toward these goals and target the groups most in need (4,5). Published national statistics† from 18 states and the District of Columbia indicate 50.9% retained in care, and 43.4% virally suppressed; however, these proportions are among persons living with HIV and are not directly comparable to the Georgia proportions, which represent only those persons with newly diagnosed HIV.

Disease stage at diagnosis has not been studied as an indicator of viral suppression. In this study, prevalence of viral suppression ≤15 months after diagnosis was significantly lower among those with stage 1 and 2 disease at diagnosis, compared with stage 3. Because 1) national HIV treatment guidelines were changed to recommend ART at all disease stages after this study’s analysis period, and 2) a recent survey of clinicians at HIV treatment centers in two states, conducted before guideline changes, revealed that only 14% would initiate ART regardless of CD4+ count (6), the lower prevalence of viral suppression among patients with an earlier disease stage at diagnosis likely resulted, in part, from fewer patients starting ART during early stages of disease. At the time of the study, the guidelines recommended treatment for persons with stage 2 disease, and the results indicate that recommended treatment for these persons was not fully implemented. Adherence to medication regimens might also be better among persons with more advanced disease, compared with those with subclinical disease (1,2). Similar to other recent studies, this report also found lack of viral suppression occurring more commonly among young persons and blacks (7–9). In addition to closing these gaps, an opportunity exists for improving viral suppression among those diagnosed at an early disease stage as guidelines for wider initiation of ART are implemented.

The findings in this report are subject to at least three limitations. First, data might have been incomplete as a result of underreporting, laboratory tests performed in other jurisdictions that might not be reported to DPH, incomplete report forms, patients lost to follow-up, or patients accessing HIV treatment outside Georgia. Second, the definition of “retained in care” might exclude patients who were tested outside the specified timeframe but are retained in care and patients who might receive care without laboratory tests. Finally, it was not possible to assess ART use or adherence.

Early diagnosis of HIV infection allows for timely interventions to achieve viral suppression, which benefits patients by improving their health status and the community by reducing HIV transmission (1). However, this study found that, even among persons retained in care, earlier diagnosis correlates with lower viral suppression. Not only were persons with stage 1 disease at diagnosis less likely to have viral suppression than those at stage 3 (as would be expected because ART was not recommended for stage 1 disease in 2010), but patients with stage 2 disease were less likely to have viral suppression than those with stage 3 disease, even though ART was recommended for both stages. It is now recommended that persons diagnosed with early disease are initiated on ART; as new guidelines are implemented, the prevalence of viral suppression should increase among this population from the baseline rate found by this study. These findings can be used to emphasize the importance of implementing the guidelines by expanding treatment to persons at all disease stages to close the gap in viral suppression among persons diagnosed at disease stages 1 and 2, and of assessing the impact of the new treatment guidelines; if no improvements in viral suppression among persons with stage 1 and 2 disease are observed in 2013, additional studies could determine if prescribing practices have not changed, or if there are other reasons for the poor suppression. All state or local health departments should monitor the continuum of care for persons living with HIV in their jurisdiction to determine care and treatment needs and evaluate public health interventions and implementation of treatment guidelines. Health-care providers and community-based organizations should implement the new treatment guidelines by initiating ART at all disease stages and inform patients about the benefits of earlier initiation of, and adherence to, ART for viral suppression at all disease stages.

What is already known on this topic?

Efforts are ongoing on national and local levels to promote testing for human immunodeficiency virus (HIV) infection, identify those with acute infection, link and retain persons living with HIV in medical care, and achieve higher rates of viral suppression. Disparities in adherence and viral suppression have been examined previously; however, disease stage at diagnosis has not been assessed as an indicator of viral suppression.

What is added by this report?

In a multivariate analysis of patients with newly diagnosed HIV infection in Georgia during 2010, disease stage at diagnosis was a statistically significant indicator of viral suppression among those retained in care, with the prevalence of viral suppression decreasing with earlier disease stage at diagnosis; fewer persons with stage 1 disease (prevalence ratio = 0.8; 95% confidence interval = 0.7–0.9) and stage 2 disease (prevalence ratio = 0.9; 95% confidence interval = 0.8–1.0) achieved viral suppression, compared with persons with stage 3 disease at diagnosis.

What are the implications for public health practice?

It is now recommended that persons diagnosed with early disease are initiated on antiretroviral therapy. As new HIV treatment guidelines are implemented, the prevalence of viral suppression should increase among this population from the baseline rate found by this study. These findings can be used to emphasize the importance of implementing and assessing the impact of the new guidelines. If no improvements in viral suppression among persons with stage 1 disease are observed in 2013, additional studies could determine if prescribing practices have not changed or if there are other reasons for the poor suppression.

## Figures and Tables

**TABLE 1:** Prevalence of viral suppression among persons aged ≥13 years who had HIV infection diagnosed in 2010 and were retained in care* (N = 1,340), by selected characteristics — Georgia†

Characteristic	Persons with diagnosed HIV		Viral suppression	
	
	No.	%	No.	%
**Overall**	**1,340**	**100.0**	**958**	**71.5**
**Race/Ethnicity**				
Black	720	53.7	456	63.3
White	149	11.1	116	78.2
Hispanic	78	5.8	61	77.9
Other/Unknown	393	29.3	325	82.7
**Age group (yrs)** §				
13–24	255	19.0	144	56.5
25–34	366	27.3	258	70.5
35–44	307	22.9	229	74.6
45–54	290	21.6	228	78.6
≥55	122	9.0	99	81.2
**Disease stage at diagnosis** ¶				
1	211	15.8	136	64.5
2	336	25.1	243	72.3
3	375	28.0	302	80.5
Unknown	418	31.2	277	66.3
**Transmission category** **				
Male	1,033	77.1 (100.0)	740	71.6
Male-to-male sexual contact	638	47.6 (61.7)	418	65.5
Injection drug use	30	2.3 (3.0)	20	65.3
Male-to-male sexual contact and injection drug use	15	1.1 (1.5)	10	66.7
Heterosexual contact	46	3.4 (4.4)	35	77.4
Other/Unknown††	304	22.7 (29.4)	257	84.5
Female	307	22.9 (100.0)	218	71.0
Injection drug use	43	3.2 (13.4)	33	78.6
Heterosexual contact	208	15.6 (67.9)	142	67.9
Other/Unknown††	56	4.2 (18.3)	43	76.8

* Retained in care defined as having at least two laboratory tests (CD4+ or viral load) at least 3 months apart during 4–15 months after diagnosis.

† Among persons residing in Georgia and having a positive Western Blot or viral load based on surveillance data reported during 2010. Viral suppression defined as viral load <200 on the basis of most recent test during 4–15 months after diagnosis. Only includes persons with their sex recorded at birth.

§ Age based on the person’s age at year’s end 2010.

¶ Based on first recorded CD4+ cell count ≤3 months after diagnosis. Defined as stage 1: CD4+ count ≥500 cells/*μ*L or CD4+ percentage of total lymphocytes ≥29; stage 2: CD4+ count of 200–499 cells/*μ*L or CD4+ percentage of total lymphocytes of 14–28; and stage 3: CD4+ count of <200 cells/*μ*L or CD4+ percentage of total lymphocytes of <14.

** Data by transmission category have been statistically adjusted to account for missing transmission category. Nested percentages indicate percentages among newly diagnosed persons of specified sex.

†† Includes hemophilia, blood transfusion, perinatal exposure, or risk factor unknown or not indicated.

**TABLE 2:** Indicators of viral suppression among persons aged ≥13 years who had HIV infection diagnosed in 2010 and were retained in care* (N = 1,340), by selected characteristics — Georgia†

Characteristic	Prevalence ratio	(95% CI)	p value
**Race/Ethnicity**			
Black	0.9	(0.8–0.9)	<0.01
White (reference group)	1.0		
Hispanic	1.0	(0.9–1.2)	0.70
Other/Unknown	1.1	(1.0–1.2)	0.07
**Age group (yrs)** §			
13–24	0.8	(0.7–0.9)	<0.01
25–34	0.9	(0.7–1.0)	0.17
35–44	0.9	(0.8–1.0)	0.23
45–54	0.9	(0.9–1.1)	0.33
≥55 (reference group)	1.0		
**Disease stage at diagnosis** ¶			
1	0.8	(0.7–0.9)	<0.01
2	0.9	(0.8–1.0)	0.02
3 (reference group)	1.0		
Unknown	0.8	(0.8–0.9)	<0.01
**Sex**			
Male	1.0		
Female	1.0	(0.9–1.1)	0.97

**Abbreviation:** CI = confidence interval.

* Retained in care defined as having at least two laboratory tests (CD4+ or viral load) at least 3 months apart during 4–15 months after diagnosis.

† Among persons residing in Georgia and having a positive Western Blot or viral load based on surveillance data reported during 2010. Viral suppression defined as viral load <200 on the basis of most recent test during 4–15 months after diagnosis. Only includes persons with their sex recorded at birth.

§ Age based on the person’s age at year’s end 2010.

¶ Based on first recorded CD4+ cell count ≤3 months after diagnosis. Defined as stage 1: CD4+ count ≥500 cells/*μ*L or CD4+ percentage of total lymphocytes ≥29; stage 2: CD4+ count of 200–499 cells/*μ*L or CD4+ percentage of total lymphocytes of 14–28; and stage 3: CD4+ count of <200 cells/*μ*L or CD4+ percentage of total lymphocytes of <14.

## Footnotes

* Reporting disease; confidentiality; reporting required of pharmacists; immunity from liability as to information supplied; notification of potential bioterrorism, O.C.G.A. §31-12-2(b) (2010).

† Available at http://www.cdc.gov/hiv/pdf/2011_monitoring_hiv_indicators_hssr_final.pdf.

## References

[1] US Department of Health and Human Services (2013). Panel on Antiretroviral Guidelines for Adults and Adolescents. Guidelines for the use of antiretroviral agents in HIV-infected adults and adolescents.

[2] Gao X, Nau DP, Rosenbluth SA, Scott V, Woodward C (2000). The relationship of disease severity, health beliefs and medication adherence among HIV patients. AIDS Care.

[3] CDC (2013). HIV surveillance report, 2011.

[4] White House Office of National AIDS Policy (2010). National HIV/AIDS strategy for the United States.

[5] CDC (2013). Monitoring selected national HIV prevention and care objectives by using HIV surveillance data—United States and 6 U.S. dependent areas—2010. HIV Surveillance Supplemental Report.

[6] Kurth AE, Mayer K, Beauchamp G (2012). Clinician practices and attitudes regarding early antiretroviral therapy in the United States. J Acquir Immune Defic Syndr.

[7] Hadland SE, Milloy MJ, Kerr T (2012). Young age predicts poor antiretroviral adherence and viral load suppression among injection drug users. AIDS Patient Care STDs.

[8] Hall HI, Frazier EL, Rhodes P (2013). Differences in human immunodeficiency virus care and treatment among subpopulations in the United States. JAMA Intern Med.

[9] Muthulingam D, Chin J, Hsu L, Scheer S, Schwarcz S (2013). Disparities in engagement in care and viral suppression among persons with HIV. J Acquir Immune Defic Syndr.

